# Smoking and caffeine consumption: a genetic analysis of their association

**DOI:** 10.1111/adb.12391

**Published:** 2016-03-30

**Authors:** Jorien L. Treur, Amy E. Taylor, Jennifer J. Ware, Michel G. Nivard, Michael C. Neale, George McMahon, Jouke‐Jan Hottenga, Bart M. L. Baselmans, Dorret I. Boomsma, Marcus R. Munafò, Jacqueline M. Vink

**Affiliations:** ^1^ Department of Biological Psychology VU University Amsterdam Amsterdam The Netherlands; ^2^ EMGO+ Institute for Health and Care Research VU University Medical Center Amsterdam The Netherlands; ^3^ UK Centre for Tobacco and Alcohol Studies, School of Experimental Psychology University of Bristol Bristol UK; ^4^ MRC Integrative Epidemiology Unit at the University of Bristol Bristol UK; ^5^ School of Social and Community Medicine University of Bristol Bristol UK; ^6^ Neuroscience Campus Amsterdam VU University Medical Center Amsterdam The Netherlands; ^7^ Virginia Institute for Psychiatric and Behavioral Genetics Virginia Commonwealth University Richmond VA USA

**Keywords:** ALSPAC, caffeine, LD‐score regression, Mendelian randomization, smoking, twin modelling

## Abstract

Smoking and caffeine consumption show a strong positive correlation, but the mechanism underlying this association is unclear. Explanations include shared genetic/environmental factors or causal effects. This study employed three methods to investigate the association between smoking and caffeine. First, bivariate genetic models were applied to data of 10 368 twins from the Netherlands Twin Register in order to estimate genetic and environmental correlations between smoking and caffeine use. Second, from the summary statistics of meta‐analyses of genome‐wide association studies on smoking and caffeine, the genetic correlation was calculated by LD‐score regression. Third, causal effects were tested using Mendelian randomization analysis in 6605 Netherlands Twin Register participants and 5714 women from the Avon Longitudinal Study of Parents and Children. Through twin modelling, a genetic correlation of *r*0.47 and an environmental correlation of *r*0.30 were estimated between current smoking (yes/no) and coffee use (high/low). Between current smoking and total caffeine use, this was *r*0.44 and *r*0.00, respectively. LD‐score regression also indicated sizeable genetic correlations between smoking and coffee use (*r*0.44 between smoking heaviness and cups of coffee per day, *r*0.28 between smoking initiation and coffee use and *r*0.25 between smoking persistence and coffee use). Consistent with the relatively high genetic correlations and lower environmental correlations, Mendelian randomization provided no evidence for causal effects of smoking on caffeine or vice versa. Genetic factors thus explain most of the association between smoking and caffeine consumption. These findings suggest that quitting smoking may be more difficult for heavy caffeine consumers, given their genetic susceptibility.

## Introduction

A recent study in two large European cohorts (one Dutch and one British) showed a strong positive correlation between smoking behaviour and caffeine consumption (Treur *et al*. 2016). When analyzing coffee, tea, cola and energy drinks separately, the strongest associations were found for coffee. Others have also demonstrated that smoking is associated with an increased consumption of coffee (Swanson, Lee, & Hopp 1994; Freedman *et al*. 2012) and of total caffeine (Hewlett & Smith 2006).

Smoking and caffeine consumption are both influenced by genetic factors. In a Dutch twin study, 44% of individual differences in smoking initiation were explained by genetic factors, while for nicotine dependence, this was 75% (Vink, Willemsen, & Boomsma 2005). The heritability estimates for coffee/caffeine use range from 36% to 77% (see overview in Vink, Staphorsius, & Boomsma 2009). Strong associations between smoking and caffeine use may therefore be the result of shared genetic and/or environmental factors. The bivariate twin model estimates genetic and environmental influences on two traits and on their overlap by comparing the resemblance in monozygotic (MZ) twin pairs to the resemblance in dizygotic (DZ) twin pairs. Few studies have applied this model to smoking and caffeine consumption. In American male twin pairs from the ‘Twin Registry of White male World War II veterans’, the observational association between number of cigarettes smoked per day, number of alcoholic drinks per week and number of cups of coffee per day was completely due to genetic factors (Swan, Carmelli, & Cardon 1996), while both genetic and non‐shared (unique) environmental factors contributed to the association between heavy smoking and heavy coffee drinking (Swan, Carmelli, & Cardon 1997). In men and women from the ‘Virginia Twin Registry’, number of cigarettes smoked per day, alcoholic drinks per week and total caffeine consumption were associated because of genetic and unique environmental factors (Hettema, Corey, & Kendler 1999). In male twin pairs from the same registry, the common environment that is shared by twins explained the correlation between caffeine consumption and cigarettes per day in adolescence. As participants aged, these common environmental influences gradually decreased to zero, and genetic influences increased (Kendler *et al*. 2008).

Recently, a novel technique to estimate genetic correlation between two traits, LD (linkage disequilibrium)‐score regression (Bulik‐Sullivan *et al*. 2015b), was developed. This method utilizes the effect size estimates of all included single nucleotide polymorphisms (SNPs) in genome‐wide association (GWA) meta‐analyses, to estimate genetic correlation between two traits. When calculating correlations among 25 phenotypes (ranging from schizophrenia to coronary artery disease), results were similar to genetic correlations estimated with individual genotype data (Bulik‐Sullivan *et al*. 2015a). To our knowledge, LD‐score regression has not yet been applied to GWA meta‐analyses on smoking (TAG 2010) and caffeine use (Cornelis *et al*. 2014).

The presence of both genetic and environmental correlations is consistent with causal effects underlying the association between traits (De Moor *et al*. 2008). Experimental work in animals and humans has provided evidence for causal effects of smoking on caffeine use (Joeres *et al*. 1988; Langmann *et al*. 2000; Benowitz, Peng, & Jacob 2003) and of caffeine use on smoking (Shoaib *et al*. 1999; Gasior *et al*. 2002; Rezvani *et al*. 2013). Mendelian randomization (MR) analysis can be employed to test causality (Davey Smith & Ebrahim 2003; Palmer *et al*. 2012; Davey Smith & Hemani 2014). MR utilizes one or several genetic variants robustly associated with a certain trait as an ‘instrument’, or proxy, for that same trait. Because of the random nature of genetic assortment, variants that are associated with a particular trait should not be associated with confounding factors. Furthermore, outcome measures cannot affect the genes that an individual is born with, removing the possibility of reverse causation. Bidirectional MR, where the effect of a genetic variant for heaviness of smoking (TAG 2010) on caffeine consumption, and the effect of eight genetic variants for caffeine consumption (Cornelis *et al*. 2014) on smoking behaviour, is tested, could unravel a possible causal association between smoking and caffeine.

We used these three methods to clarify the nature of the association between smoking and caffeine use. First, in a large sample of 10 368 twins from the Netherlands Twin Register (NTR), bivariate genetic models were applied to data on smoking and caffeine consumption. Second, genetic correlation between smoking and caffeine was computed with LD‐score regression, utilizing data from recent GWA meta‐analyses. Third, in a sample of 6605 participants from the NTR and 5714 from the Avon Longitudinal Study of Parents and Children (ALSPAC), causal effects were tested using bidirectional MR.

## Materials and Methods

**Figure 1:**
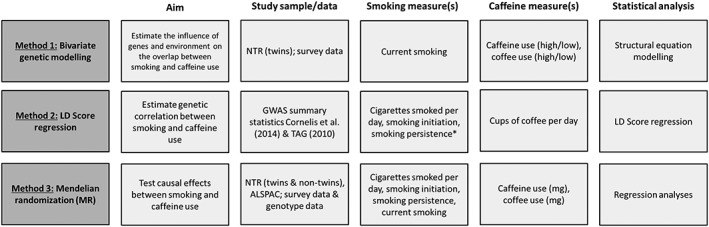
Overview of three methods employed to investigate the association between smoking and caffeine consumption. The asterisk (*) means that the original measure from TAG (2010) was smoking cessation (0 = current smoking, 1 = former smoking); this was defined here as smoking persistence (0 = former smoking, 1 = current smoking). NTR, Netherlands Twin Register; ALSPAC, Avon Longitudinal Study of Parents and Children; TAG, Tobacco, Alcohol and Genetics Consortium; GWAS, genome‐wide association studies

Figure 1 provides an overview of the three methods, with the corresponding aims, study samples/data, smoking measures, caffeine measures and statistical analyses. More detailed information on each of the approaches is provided as follows.

### Study samples/data

#### The Netherlands Twin Register

The NTR is an ongoing longitudinal study of Dutch twins and their family members (Willemsen *et al*. 2013). The 10th NTR survey sent in 2013–2014 contained questions on an extensive list of caffeinated and decaffeinated drinks (including coffee, tea, cola and energy drinks) and on smoking behaviour. The fifth NTR survey sent in 2000 contained questions on (caffeinated) coffee consumption and smoking. For those who did not complete the 10th survey, data from the fifth survey were included. NTR participants were included in the bivariate twin modelling analyses (method 1) and in the MR analyses (method 3).

A total of 10 368 twins with data on smoking and coffee use were available for bivariate modelling {mean age = 32.5 [standard deviation (SD) 14.5], 68.1% female, 6866 twins from complete pairs and 3502 twins from incomplete pairs}. Of this total, 1425 twins were monozygotic male (MZM), 907 dizygotic male (DZM), 3541 monozygotic female (MZF), 1948 dizygotic female (DZF) and 2547 dizygotic opposite sex (DOS). For 8060 twins, there were data on smoking and total caffeine use (mean age = 33.7 [SD 15.3], 69.0% female, 4778 twins from complete pairs and 3282 twins from incomplete pairs). In this group, there were 1089 MZM, 677 DZM, 2837 MZF, 1498 DZF and 1959 DOS twins.

For MR analysis, phenotype and genotype data were available in up to 6605 NTR participants (mean age = 42.7 [SD 16.7], 66.4% female), of which 1415 had data on smoking and coffee only.

#### The Avon Longitudinal Study of Parents and Children

The Avon Longitudinal Study of Parents and Children is a prospective cohort study that recruited 14 541 pregnant women who resided in the county of Avon in the UK and who had expected delivery dates ranging between 1 April 1991 and 31 December 1992. The ALSPAC Ethics and Law Committee and the Local Research Ethics Committees gave ethical approval for the study. An elaborate account of ALSPAC and its methods is given elsewhere (Boyd *et al*. 2012; Fraser *et al*. 2013). Questions on smoking and caffeinated and decaffeinated coffee, tea and cola consumption were asked in surveys sent to the mothers during pregnancy at 18 weeks gestation, 32 weeks gestation and after delivery when the child was aged 2, 47, 85, 97 and 145 months. Analyses were performed for all timepoints, but only the results from when the child was 47 months are reported. This specific timepoint was selected because the sample size was large and because during and immediately after pregnancy, smoking behaviour and caffeine use may be different. Please note that the study website contains details of all the data that are available through a fully searchable data dictionary (www.bris.ac.uk/alspac/researchers/data‐access/data‐dictionary).

Participants registered at ALSPAC were included in the MR analyses (method 3). Phenotype and genotype data of 5714 participants were available (mean age = 33.4 [SD 4.5]).

#### Genotype data

Single nucleotide polymorphism data were available from genome‐wide SNP arrays. In the NTR sample, these data were collected through several projects between 2004 and 2008. Adult participants (18+) who had participated in NTR research at least once were selected for genotyping. Full details on the data collection and genotyping methods in NTR can be found elsewhere (Willemsen *et al*. 2010; Nivard *et al*. 2014). In ALSPAC, DNA was extracted from blood samples that were collected from 10 000 mothers during their normal antenatal care. More details on the collection of DNA and genotyping methods in ALSPAC are provided elsewhere (Jones *et al*. 2000; Hinds *et al*. 2013). Genotype data were utilized in the MR analyses (method 3).

Genetic risk for smoking was reflected by SNP rs1051730, located in the *CHRNA3* gene at chromosome 15 and robustly and consistently associated with smoking heaviness (TAG 2010; Thorgeirsson *et al*. 2010). This SNP is in high LD with rs16969968 (TAG 2010). For caffeine use, a genetic risk score was created based on eight SNPs that reached genome‐wide significance in their association with coffee consumption in a large meta‐analysis (Cornelis *et al*. 2014). The number of coffee consuming increasing alleles at each locus was summed across all eight variants for each individual. Alleles were weighted according to the magnitude of the effect size (β) for coffee consumption, taken from the recent meta‐analysis by Cornelis *et al*. (2014). Table 1 provides more detailed information on all SNPs, including the risk alleles, frequencies of these risk alleles and effect sizes.

**Table 1:** SNPs utilized in Mendelian randomization analysis.

SNP	Chr	Closest gene	RA/NRA	Effect size	RAF		Genotyped/imputed		Imputation quality	
					NTR	ALSPAC	NTR	ALSPAC	NTR	ALSPAC
Smoking heaviness										
rs1051730	15	*CHRNA3*	A/G	1.03	0.32	0.33	G	G	—	—
Coffee consumption										
rs1260326	2	*GCKR*	C/T	0.04	0.63	0.60	G	G	—	—
rs1481012	4	*ABCG2*	A/G	0.06	0.89	0.90	G	G	—	—
rs6968554	7	*AHR*	G/A	0.13	0.64	0.64	G	I	—	0.99
rs7800944	7	*MLXIPL*	C/T	0.05	0.27	0.29	I	I	0.79	0.97
rs17685	7	*POR*	A/G	0.07	0.26	0.28	G	G	—	—
rs6265	11	*BDNF*	C/T	0.05	0.80	0.81	G	G	—	—
rs2472297	15	*CYP1A1*	T/C	0.15	0.27	0.27	G	G	—	—
rs9902453	17	*EFCAB5*	G/A	0.04	0.49	0.45	G	I	—	0.99

Effect sizes represent β coefficients and were obtained from TAG (2010) and Cornelis *et al*. (2014).

ALSPAC, Avon Longitudinal Study of Parents and Children; Chr, chromosome; RA, risk allele (smoking or coffee consumption increasing allele); NRA, non‐risk allele; NTR, Netherlands Twin Register; RAF, risk allele frequency in the total study sample of ALSPAC and NTR participants; SNP, single nucleotide polymorphism.

### Smoking measures

All NTR and ALSPAC participants were classified as current smokers, former smokers or never smokers. A variable on smoking heaviness (cigarettes smoked per day) was available for current smokers in both cohorts. A more detailed explanation of these variables is available in a publication on the observational associations between smoking and caffeine consumption in NTR and ALSPAC (Treur *et al*. 2016). For the current paper, variables were defined that reflect smoking initiation (0 = never smokers, 1 = former and current smokers), current smoking (0 = never and former smokers, 1 = current smokers) and smoking persistence (0 = former smokers, 1 = current smokers).

### Caffeine measures

In both NTR and ALSPAC, questions were asked about the consumption of caffeinated coffee, tea and cola, while in the NTR, an additional question on energy drinks was included. From these questions, daily total caffeine consumption (in mg) and daily caffeine consumption through coffee (in mg) were calculated for all participants. More details on the making of these variables are given elsewhere (Treur *et al*. 2016). For bivariate twin modelling (method 1; NTR data only), a dichotomous variable was created where 1 SD above the mean was chosen as a cut‐off point, distinguishing ‘low’ from ‘high’ coffee users (0 = low [≤1 SD above the mean, *N* = 8599], 1 = high [>1 SD above the mean, *N* = 1769]) and ‘low’ from ‘high’ total caffeine users (*N* = 6863 and *N* = 1197, respectively) This cut‐off point was determined for men and women separately.

### Statistical analyses

#### Bivariate genetic modelling

The bivariate twin model estimates the influence of additive genetic effects (A), common environmental effects shared by twins from the same family (C) and unique environmental effects (E) on smoking and caffeine use, as well as how much of the correlation between smoking and caffeine use is due to A, C and E. Briefly, MZ twins are 100% genetically similar, while DZ twins share ~50% of their segregating genes; both types of twins may share their environment. In the case of one trait, a higher resemblance between MZ twins than between DZ twins indicates an influence of additive genetic factors (A). If the correlation between DZ twins is greater than half the correlation between MZ twins, the common environment that is shared by both twins (C) is also of influence. When the correlation between MZ twins is lower than 1, this must be due to unique environmental factors (E). The influence of genes and environment on the correlation between smoking and caffeine (bivariate) is deduced from the correlation between smoking in twin 1 and caffeine in twin 2. When this ‘cross‐correlation’ is higher in MZ than in DZ twin pairs, an influence of A is implied. When the DZ cross‐correlation is greater than half the MZ cross‐correlation, the influence of C is suggested. When the MZ cross‐correlation is lower than the correlation between smoking and caffeine in one person, an influence of E is implied. For more elaborate descriptions and comparable bivariate twin designs, see, e.g. Kiecolt, Aggen, & Kendler (2013); Posthuma *et al*. (2003) and Poelen *et al*. (2008).

To estimate genetic and environmental influences, bivariate structural equation modelling was performed in OpenMx (Boker *et al*. 2011). There were two models, one with current smoking (0 = never and former smokers, 1 = current smokers) and coffee use (0 = low, 1 = high) and one with current smoking and total caffeine use (0 = low, 1 = high). In these so‐called liability threshold models, an underlying liability resulting from genetic and environmental factors is assumed. A threshold divides individuals into current smokers and non‐current smokers and into high and low caffeine users. The thresholds depend on the prevalence of current smoking and high caffeine use, respectively (Falconer & Mackay 1996; Wray & Visscher 2015). Age was included as a moderator on the thresholds, allowing prevalence to differ with age (categories: <20, 20–24, 25–34, 35–44, 45–54 and ≥55 years).

The first step of genetic modelling was to fit a bivariate saturated model to data from five sex‐by‐zygosity groups (MZM, DZM, MZF, DZF and DOS twin pairs). Next, the effects of A, C and E on smoking, coffee/caffeine and the genetic and environmental correlations were estimated in a bivariate ACE model. Several constraints were imposed during model fitting, which are described in the Results section. The fit of submodels was tested with likelihood ratio tests, following a *χ* ^2^ distribution where the amount of d.f. (degrees of freedom) is equal to the difference in d.f. of the two models. Constraints were retained when they did not significantly deteriorate the fit (*P*‐value ≥0.05).

#### LD‐score regression

Genome‐wide association meta‐analysis results were available for cigarettes smoked per day, smoking initiation (0 = never smokers, 1 = former and current smokers) and smoking cessation (0 = current smoking, 1 = former smoking) (TAG 2010) and for cups of coffee per day (Cornelis *et al*. 2014). Smoking cessation was defined as smoking persistence (0 = former smokers, 1 = current smokers) by multiplying the genetic correlation by −1. The meta‐analyses on smoking and coffee included GWA studies of 16 and 28 population‐based samples of European ancestry, including up to 46 481 individuals and 91 462 individuals, respectively. Findings are thus not restricted, or specific, to one single population.

Genetic correlations were estimated using LD‐score regression (Bulik‐Sullivan *et al*. 2015a). The intuitive concept behind this technique is that for highly polygenic traits, SNPs that tag many neighbouring SNPs due to strong LD have a higher chance of tagging a causal locus. In contrast, SNPs that are in relatively weak LD with their neighbours tag fewer causal loci. One can therefore formulate the expected effect size for a SNP in a GWAS as a function of the degree of LD, sample size in a GWAS, number of SNPs considered and the heritability. To estimate genetic correlation, the effect size estimates of all SNPs included in GWAS of two phenotypes of interest are utilized. First, the association between a particular SNP and phenotype 1 (represented by a Z score) is multiplied by the association between that same SNP and phenotype 2. Second, the product thereof is regressed on the LD that the SNP has with all neighbouring SNPs (i.e. the LD score). As such, it is possible to estimate genetic correlation between two traits solely based on observed summary statistics. We used precomputed LD scores based on meta‐analyses of individuals of European ancestry that are publicly available (from: https://github.com/bulik/ldsc). LD‐score regression can be utilized even when there is sample overlap, because effect size inflation due to sample overlap will equally impact all SNPs, regardless of their LD score, and inflation due to sample overlap inflates the intercept, not the slope.

#### Mendelian randomization analysis

**Figure 2:**
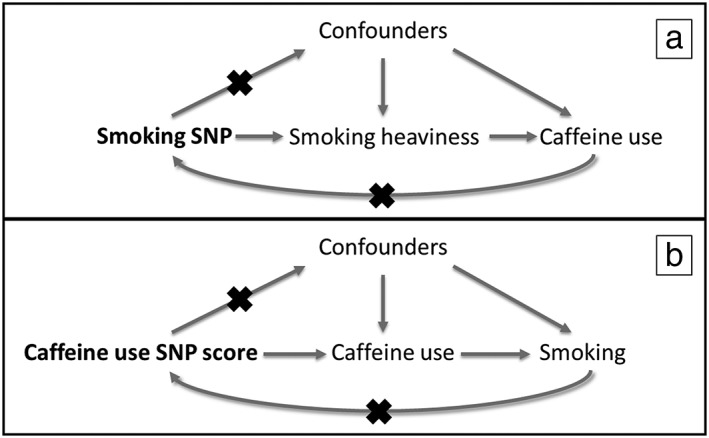
Principle of bidirectional Mendelian randomization (MR) applied to the association between smoking and caffeine use. Smoking SNP = rs1051730; caffeine use SNP score = genetic risk score of caffeine use based on eight SNPs (rs1260326, rs1481012, rs6968554, rs7800944, rs17685, rs6265, rs2472297 and rs9902453). (a) Under a causal effect of smoking on caffeine use, the smoking SNP should, through its effect on smoking heaviness, increase caffeine use (among smokers only). (b) Under a causal effect of caffeine use on smoking, the caffeine use SNP score should, through its effect on caffeine use, increase smoking heaviness, smoking initiation and/or smoking cessation. MR rules out reverse causation [represented by the arrow going from caffeine use to the smoking SNP in (a) and from smoking to the caffeine use SNP score in (b)]. An important aspect of MR is that the genotype in question should not be associated with confounders [represented by the arrow going from the smoking SNP to confounders in (a) and from the caffeine use SNP score to confounders in (b)]

By measuring genetic variants strongly predictive of smoking and caffeine use instead of these behaviours themselves, MR minimizes effects of confounding and reverse causation (Fig. 2). In Stata (version 9.0; StataCorp LP, College Station, TX, USA), regression analyses were first carried out between the smoking SNP and smoking behaviour and between the caffeine use risk score and caffeine consumption to test their instrumental value. The smoking SNP should be associated with number of cigarettes smoked per day, and the caffeine use risk score should be associated with amount of caffeine consumed per day. Next, the smoking SNP was associated with caffeine use (Fig. 2a) and the caffeine use SNP score with smoking (Fig. 2b), to test causal effects. Data from NTR and ALSPAC cohorts were pooled to increase power and corrected for age (continuous), gender (only relevant in NTR, 0 = male, 1 = female) and sample (0 = NTR, 1 = ALSPAC). For NTR participants, analyses were corrected for family clustering by utilizing the robust cluster option in Stata. We also tested whether genetic risk variants for smoking and caffeine were associated with potential confounding factors (educational attainment and social class).

## Results

### Bivariate genetic modelling

In the whole twin sample, 11.6% was aged <20 years, 30.1% 20–24 years, 22.3% 25–34 years, 18.2% 35–44 years, 7.0% 45–54 years and 10.8% ≥55 years. The association of age with smoking and caffeine prevalence was estimated separately in men and women by a regression of age (β) on the thresholds in the liability model. Prevalences were equal across twin birth order but not across zygosity group. On this saturated model with 46 free parameters (Table 2‐A; model 1), we applied several constraints. Dropping age from the model resulted in a significant deterioration of the model fit (model 2), so it was kept in. The modelled prevalence of current smoking and high coffee/total caffeine use across age groups are depicted in Table S1. The effect of age on smoking and coffee was different for men than for women (models 3 and 4), so it was not constrained across gender. Within person correlations between smoking and coffee (cross‐trait‐within twin) and cross‐correlations between smoking in twin 1 and coffee in twin 2 (cross‐trait cross‐twin correlations) could be constrained across twin birth order (models 5 and 6). No differences in twin resemblance were found between men and women (model 7). For current smoking and total caffeine use, the same constraints were allowed (Table 2‐B; models 1 till 7). Twin correlations from the best‐fitting saturated models are given in Table 3.

**Table 2:** Structural equation models to explore additive genetic (A), common environmental (C) and unique environmental (E) influences on current smoking and caffeine use and on their overlap.

A: Current smoking and coffee use (*N* = 10 368)	Estimated parameters	−2LL	d.f.	Compared with	*χ* ^2^	*P*‐value
1. Saturated five‐group model	46	17 021.62	20 690	—	—	—
2. β's covariate dropped	42	17 850.72	20 694	1	829.11	<0.001
3. Thresholds/β's constrained across sex for smoking	41	17 041.36	20 695	1	19.74	<0.001
4. Thresholds/β's constrained across sex for coffee	41	17 126.56	20 695	1	104.95	<0.001
5. Cross‐trait‐within twin correlation constrained across twin birth order	41	17 027.16	20 695	1	5.55	0.35
6. Cross‐trait cross‐twin correlation constrained across twin birth order	36	17 032.36	20 700	5	5.2	0.39
**7. Correlations MZM = MZF + correlations DZM = DZF = DOS**	**24**	**17 043.87**	**20 712**	**6**	**11.5**	**0.49**
8. ACE model	25	17 046.54	20 715	1	24.92	0.47
9. C for coffee dropped	24	17 048.9	20 716	8	2.36	0.12
10. C for smoking dropped	23	17 048.9	20 717	9	0	1.0
**11. C for overlap dropped**	**22**	**17 048.99**	**20 718**	**10**	**0.09**	**0.76**
12. A for coffee dropped	21	17 256.83	20 719	11	207.84	<0.001
13. A for smoking dropped	21	17 148.73	20 719	11	99.74	<0.001
14. A for overlap dropped	21	17 143.17	20 719	11	94.18	<0.001
15. E for overlap dropped	21	17 064.68	20 719	11	15.69	<0.001
B: Current smoking and total caffeine use (*N* = 8060)	Estimated parameters	−2LL	d.f.	Compared with	*X* ^2^	*P*‐value
1. Saturated five‐group model	46	12 194.18	16 074	—	—	—
2. β's covariate dropped	42	12 761.54	16 078	1	567.36	<0.001
3. Thresholds/β's constrained across sex for smoking	41	12 209.66	16 079	1	15.48	0.01
4. Thresholds/β's constrained across sex for total caffeine	41	12 207.36	16 079	1	13.18	0.02
5. Cross‐trait‐within twin correlation constrained across twin birth order	41	12 194.68	16 079	1	0.5	0.99
6. Cross‐trait cross‐twin correlation constrained across twin birth order	36	12 195.81	16 084	5	1.13	0.95
**7. Correlations MZM = MZF + correlations DZM = DZF = DOS**	**24**	**12 207.23**	**16 096**	**6**	**11.42**	**0.49**
8. ACE model	25	12 209.95	16 099	1	15.77	0.83
9. C for total caffeine dropped	24	12 211.23	16 100	8	1.29	0.26
10. C for smoking dropped	23	12 211.23	16 101	9	0	0.98
11. C for overlap dropped	22	12 211.34	16 102	10	0.1	0.75
12. A for total caffeine dropped	21	12 311.73	16 103	11	100.39	<0.001
13. A for smoking dropped	21	12 273.89	16 103	11	62.55	<0.001
14. A for overlap dropped	21	12 240.56	16 103	11	29.23	<0.001
**15. E for overlap dropped**	**21**	**12 212.95**	**16 103**	**11**	**1.62**	**0.20**

A threshold represents the prevalence of smoking or coffee/caffeine use. β = effect of age on the prevalence (threshold) of smoking or coffee/caffeine use. The best‐fitting models are depicted in bold.

DOS, dizygotic opposite sex twin pairs; DZF, dizygotic female twin pairs; DZM, dizygotic male twin pairs; MZF, monozygotic female twin pairs; MZM, monozygotic male twin pairs.

**Table 3:** Twin correlations for current smoking and coffee use (*N* = 10 368) and for current smoking and total caffeine use (*N* = 8060) from the best‐fitting saturated models.

	Cross‐twin within‐trait				Cross‐twin within‐trait			
	Current smoking	Coffee	Within‐twin cross‐trait	Cross‐twin cross‐trait	Current smoking	Total caffeine	Within‐twin cross‐trait	Cross‐twin cross‐trait
MZ	0.75 (0.74 to 0.78)	0.52 (0.49 to 0.59)	0.37 (0.36 to 0.37)	0.26 (0.20 to 0.27)	0.73 (0.64 to 0.80)	0.47 (0.37 to 0.49)	0.23 (0.15 to 0.30)	0.18 (0.09 to 0.20)
DZ	0.41 (0.32 to 0.50)	0.32 (0.30 to 0.35)	0.43 (0.41 to 0.45)	0.21 (0.16 to 0.29)	0.40 (0.27 to 0.51)	0.30 (0.15 to 0.43)	0.31 (0.24 to 0.38)	0.18 (0.07 to 0.29)

Cross‐twin within trait = correlation between smoking twin 1 and smoking twin 2 or coffee/caffeine twin 1 and coffee/caffeine twin 2. Within‐twin cross‐trait = correlation between smoking and coffee/caffeine in one twin. Cross‐twin cross‐trait = correlation between smoking twin 1 and coffee/caffeine twin 2.

DZ, dizygotic; MZ, monozygotic.

Next, an ACE model was fitted for current smoking and coffee use (Table 2‐A; models 8 till 15). Dropping C for coffee, for smoking, or for the overlap between coffee and smoking was permitted (models 9–11). Dropping A for coffee, for smoking, or for the overlap, and E for the overlap resulted in a significant deterioration of the fit (models 12 till 15), and these effects were thus retained. For current smoking and total caffeine use, similar submodels were applied (Table 2‐B; models 8 till 15), resulting in a best‐fitting model without any influence of C and without E for the overlap.

Table 4 depicts parameter estimates for the full ACE and best‐fitting AE models. Variation in current smoking was mostly due to additive genetic factors (A_CurrentSmoking_ = 76% [70–79%] in a bivariate model with coffee and A_CurrentSmoking_ = 74% [66–80%] with total caffeine) with the remainder being due to unique environmental factors (E_CurrentSmoking_ = 24% [19–30%] and E_CurrentSmoking_ = 26% [20–34%], respectively). About half of the variation in coffee use was due to additive genetic factors (A_Coffee_ = 53% [48–58%]) and the other half to unique environmental factors (E_Coffee_ = 47% [40–54%]). For total caffeine, results were similar (A_TotalCaffeine_ = 49% [47–58%] and E_TotalCaffeine_ = 51% [42–60%]). See Figs S1 and S2 for a graphical representation of these models, with all parameter estimates.

**Table 4:** Estimates of additive genetic (A), common environmental (C) and unique environmental (E) influences on current smoking and coffee use (*N* = 10 368) and on current smoking and total caffeine use (*N* = 8060), from the full and the best‐fitting bivariate genetic models.

	Current smoking	Coffee	Phenotypic overlap	Current smoking	Total caffeine	Phenotypic overlap
Full model						
A	0.67 (0.51 to 0.79)	0.40 (0.15 to 0.58)	0.48 (0.17 to 0.84)	0.64 (0.37 to 0.79)	0.36 (0.03 to 0.56)	0.44 (0.00 to 1.00)
C	0.08 (0.02 to 0.25)	0.12 (0.00 to 0.32)	0.24 (0.10 to 0.55)	0.08 (0.00 to 0.31)	0.12 (0.00 to 0.40)	0.35 (0.00 to 0.88)
E	0.25 (0.21 to 0.31)	0.48 (0.41 to 0.54)	0.28 (0.24 to 0.42)	0.28 (0.21 to 0.36)	0.52 (0.43 to 0.62)	0.21 (0.00 to 0.50)
Best‐fitting model						
A	0.76 (0.70 to 0.79)	0.53 (0.48 to 0.58)	0.75 (0.62 to 0.87)	0.74 (0.66 to 0.80)	0.49 (0.47 to 0.58)	1.00 (1.00 to 1.00)
C	—	—	—	—	—	—
E	0.24 (0.19 to 0.30)	0.47 (0.40 to 0.54)	0.25 (0.13 to 0.38)	0.26 (0.20 to 0.34)	0.51 (0.42 to 0.60)	—
Genetic correlation	0.47 (0.38 to 0.56)			0.44 (0.35 to 0.53)		
Unique environmental correlation	0.30 (0.15 to 0.45)			0.00 (0.00 to 0.00)		

Phenotypic overlap reflects how much of the observational association between current smoking and coffee/total caffeine is due to additive genetic (A), common environmental (C) and unique environmental (E) influences, summing up to 1.

A genetic correlation of *r*0.47 (0.38 to 0.56) was found between smoking and coffee and of *r*0.44 (0.35 to 0.53) between smoking and total caffeine. There was a unique environmental correlation of *r*0.30 (0.15 to 0.45) between smoking and coffee. The unique environmental correlation between smoking and total caffeine was *r*0.00 (0.00 to 0.00), meaning that all correlation between the two traits was attributed to other sources (namely genetic). Phenotypic overlap between current smoking and coffee was mostly due to additive genetic factors (A_CurrentSmoking‐Coffee_ = 75% [62–87%]), with some influence of unique environmental factors (E_CurrentSmoking‐Coffee_ = 25% [13–38%]). Between current smoking and total caffeine, the overlap was completely due to genetic factors.

### LD‐score regression

The genetic correlation between cigarettes per day and cups of coffee per day, as calculated by LD‐score regression, was *r*0.44 (0.14 to 0.74). This confirms the results from the bivariate twin models of the same genetic risk factors influencing current smoking and high coffee/total caffeine consumption. Between smoking initiation and cups of coffee per day, the genetic correlation was lower at *r* 0.28 (0.11 to 0.45), and between smoking persistence and cups of coffee per day, it was *r*0.25 (0.04 to 0.46).

### Mendelian randomization analysis

As expected, the caffeine use SNP score was strongly associated with a higher coffee and total caffeine consumption in mg of caffeine per day (Fig. S3‐A; β coefficient 66.5 [45.0–87.90] and 86.7 [64.4–109.1], respectively). The smoking SNP (rs1051730) was associated with more cigarettes smoked per day (Fig. S3‐B; 0.6 [0.2–1.1]), confirming previous studies.

The caffeine use SNP score was not associated with educational attainment or social class, while for the smoking SNP, there was some minor evidence for a negative association in ALSPAC only (Table S3). This association is most likely spurious, as discussed elsewhere (Taylor *et al*. under review). Briefly, Taylor *et al*. found no consistent evidence for a causal, negative effect of smoking on socio‐economic status when analysing the effect of the smoking SNP on different measures of socio‐economic status in ALSPAC and the Nord‐Trøndelag Health Study.

There was no association between the smoking SNP and caffeine consumption (total or coffee only) in current, former or never smokers (Fig. S4‐A), thus providing no support for a causal effect of smoking on caffeine. Also, none of the pooled analyses showed an association between the caffeine use SNP score and smoking behaviour, meaning that there is no evidence for a causal effect of caffeine use on smoking behaviour (Fig. S4‐B). See Tables S4 and S5 for MR analyses at all timepoints in ALSPAC.

## Discussion

The association between smoking and caffeine consumption was investigated with three methods: bivariate twin modelling, LD‐score regression and MR. All three sets of analyses pointed to a similar conclusion of shared genetic factors explaining the phenotypic overlap between smoking and caffeine use. The lack of evidence for causal effects between smoking and caffeine use could have been due to low power.

It is the first time that the association between smoking and caffeine use was investigated in Dutch twins. Our results are in line with earlier US‐based twin studies that found genetic correlation between smoking and caffeine use (Swan *et al*. 1996, 1997; Hettema *et al*. 1999; Kendler *et al*. 2008). Apart from one (Swan *et al*. 1996), these studies also found unique environmental correlation between smoking and caffeine use (Swan *et al*. 1997; Hettema *et al*. 1999; Kendler *et al*. 2008) as we did for smoking and coffee use. For smoking and total caffeine use, we did not find unique environmental correlation. In the present study, we analysed caffeine as high versus low users and smoking as current versus non‐current smokers. To demonstrate that our conclusions were not affected by the dichotomization of caffeine (which may result in a loss of statistical power), we also applied a bivariate ACE model with caffeine as a continuous measure (mg per day). The results of these analyses were very similar with the exception that there was, in addition to a genetic correlation, also a unique environmental correlation for both smoking and coffee and smoking and total caffeine (Tables S3–S5). The fit of this alternative model was poor when compared with the fully saturated model, probably because the continuous measure of caffeine was severely (right) skewed. In spite of the variation in measures of smoking and caffeine use, the present study corroborates previous twin studies. The genetic correlations of *r*0.47 (for coffee only) and *r*0.44 (for total caffeine) are very similar to those found between cigarettes per day and cups of coffee per day (*r*0.43; Swan *et al*. 1996) and between heavy smoking and heavy coffee use (*r*0.43; Swan *et al*. 1997). An important strength of our study is that it involves a large sample of >10 000 twins, making it twice as large as the biggest previous twin study (Swan *et al*. 1997). Even though the Netherlands is seen as a typical ‘coffee drinking’ country (Fredholm 2011; Ferdman 2014), the genetic underpinnings of the overlap between smoking and caffeine were similar compared with US populations.

Genetic correlations as estimated by twin modelling were remarkably similar to the genetic correlation between cigarettes per day and cups of coffee per day based on effect size estimates of two large GWA meta‐analyses. Some caution is warranted with the comparison of these two methods, however, given the difference in measures of smoking and caffeine use. A major advantage of LD‐score regression is that genetic correlation is based on data of multiple (European) populations, instead of just one (Dutch) population in the case of twin modelling. There was also evidence for an overlap between SNPs associated with cups of coffee per day and smoking initiation and smoking persistence. Genetic variants that increase coffee consumption thus also increase the chance of becoming a smoker and decrease the chance of quitting smoking once started. The latter is in agreement with observational studies finding an inverse relation between quitting smoking and coffee consumption (Sorlie & Kannel 1990; Olsen 1993; Fernandez *et al*. 1997). The present study is the first to correlate SNPs associated with smoking behaviour with SNPs associated with coffee consumption.

In Dutch and British individuals, there was no evidence for causal effects of smoking on caffeine use, or vice versa, as also suggested from the bivariate twin data, in which the unique environmental correlation was low or zero (De Moor *et al*. 2008). MR analyses may have been underpowered to pick up on causal effects. Given the study's sample size, an increase of 29 mg of caffeine with each cigarette per day would have provided evidence for a causal effect of smoking on caffeine (power of 0.80), as opposed to the increase of 5 mg that we found. Whereas each extra mg of caffeine was associated with +0.01 cigarettes per day, an increase of 0.04 would have pointed to a causal association of caffeine on smoking (Brion, Shakhbazov, & Visscher 2013). Although the eight SNPs included in the caffeine use SNP score were taken from a study looking exclusively at coffee use (Cornelis *et al*. 2014), the two most significant SNPs (rs6968554 and rs2472297) were recently also positively associated with total caffeine (coffee + tea + cola), coffee and tea, but not with cola alone (McMahon *et al*. 2014). A potential limitation of MR is pleiotropy. Under pleiotropy, one genetic variant or set of variants is associated with multiple phenotypes. It could, e.g. be the case that the caffeine use SNP score directly affects smoking (not acting through a causal effect of caffeine use on smoking), which would undermine the principle of MR. Pleiotropic effects can be minimized by selecting genetic instruments with effects that plausibly act directly on the trait in question. Also, when different (sets of) SNPs separately have the same association with the outcome of interest, it is less likely that this is due to pleiotropy (Davey Smith & Hemani 2014). In the present study, MR analyses were therefore repeated with a genetic risk score for caffeine including only rs6968554 and rs2472297. Both of these SNPs play a clear role in the metabolism of caffeine, and it is unlikely that they have a direct effect (e.g. not acting through caffeine use) on smoking. The results of these analyses were very similar to the risk score based on eight SNPs (data not shown), suggesting that pleiotropy did not affect the results.

This study was the first to combine multiple methods with the aim of unravelling the nature of the co‐morbidity between smoking and caffeine use. Our findings point to shared genetic factors underlying the association between smoking and caffeine use, not ruling out that there is an additional (smaller) influence of causal effects. At least, some of the genetic risk factors for smoking overlap with genetic risk factors for caffeine use. This finding suggests that initiating smoking may be especially undesirable for heavy caffeine users, given their genetic susceptibility to smoke more heavily or to more easily become nicotine dependent. Because smoking is likely to be initiated before heavy caffeine use is manifested, a more important implication may be that smokers who are also heavy caffeine users might, on average, find it more difficult to quit because of their genetic background. To confirm our findings and to further clarify the complex association between these (addictive) behaviours, further research is required. Especially, causal effects from smoking on caffeine or vice versa need to be explored through MR analysis in larger samples.

## Supporting information


**Table S1**. Prevalences of current smoking, high coffee use and high total caffeine use as estimated in structural equation models
**Table S2**. Twin correlations from structural equation models before constraining correlations across gender
**Table S3**. Structural equation models to explore additive genetic (A), common environmental (C) and unique environmental (E) influences on current smoking and caffeine use in mg per day, and on their overlap
**Table S4**. Twin correlations for current smoking and coffee use in mg per day (N = 10,368) and for current smoking and total caffeine use in mg per day (N = 8,060) from the best‐fitting saturated models
**Table S5**. Estimates of additive genetic (A), common environmental (C) and unique environmental (E) influences on current smoking and coffee use in mg per day (N = 10,368) and on current smoking and total caffeine use in mg per day (N = 8,060), from the full and the best‐fitting bivariate genetic models
**Table S6**. Associations between the caffeine use SNP score and the smoking SNP and confounding variables in *the Netherlands Twin Register (NTR) and the Avon Longitudinal Study of Parents and Children (ALSPAC)*
**Table S7**. Mendelian Randomization analyses between the caffeine use SNP score and daily caffeine consumption (in mg) and smoking behaviour in the *Avon Longitudinal Study of Parents and Children (ALSPAC)*
**Table S8**. Mendelian Randomization analyses between the smoking SNP and smoking behaviour and daily caffeine consumption (in mg) in the *Avon Longitudinal Study of Parents and Children (ALSPAC)*
**Figure S1**. Path estimates for bivariate genetic models on current smoking & coffee and on current smoking & total caffeine. A = additive genetic factors, C = common genetic factors, E = unique environmental factors. Both the initial models and the best‐fitting models are shown.
**Figure S2**. Path estimates for bivariate genetic models on current smoking & coffee and on current smoking & total caffeine. A = additive genetic factors, C = common genetic factors, E = unique environmental factors. Here, genetic (between A1 and A2), common environmental (between C1 and C2) and unique environmental (E1 and E2) correlations are shown. Calculation of the genetic correlation was based on the following formula: a_CurrentSmoking_ * a_CurrentSmoking‐Coffee_ / √(a^2^_CurrentSmoking_) * √( a^2^_CurrentSmoking‐Coffee_ + a^2^_Coffee_), where a_CurrentSmoking_ and a_Coffee_ represent the path loadings going from A1 to ‘Current smoking’ and from A2 to ‘Coffee’, respectively and a_CurrentSmoking‐Coffee_ represents the path loading going from A2 to ‘Current smoking’ in Figure S1. Environmental correlations were calculated in the same way. Both the initial models and the best‐fitting models are shown.
**Figure S3**. Instrumental value of the genetic risk scores. The forest plots show associations between the caffeine use SNP score and total caffeine use and caffeine from coffee in mg per day (A) and between the smoking SNP and cigarettes smoked per day (B). NTR = Netherlands Twin Register; ALSPAC = Avon Longitudinal Study of Parents and Children.
**Figure S4**. MR analyses testing causal effects. The forest plots show associations between the smoking SNP and total caffeine use and caffeine from coffee in mg per day (A) and between the caffeine use SNP score and smoking behaviour (cigarettes smoked per day, smoking persistence, smoking initiation and current smoking) (B). NTR = Netherlands Twin Register; ALSPAC = Avon Longitudinal Study of Parents and Children.

Supporting info item
