# Supplementary material for: Smoking and caffeine consumption: a genetic analysis of their association
Source: Addict Biol. 2016 Mar 30;22(4):1090–102. doi: 10.1111/adb.12391 (PMC5045318; doi:10.1111/adb.12391)
Supplement: Supplementary file 1 — Supporting info item [file ADB-22-1090-s001.docx]

**Supplement to manuscript “Smoking and caffeine consumption: A genetic analysis of their association”**

*Bivariate genetic modelling*

Prevalences of current smoking, high coffee use and high total caffeine use as estimated in structural equation models ____________________2

Twin correlations from structural equation models before constraining correlations across gender _____________________________________3

Bivariate genetic models on current smoking & coffee and on current smoking & total caffeine ________________________________________4

Bivariate genetic models on current smoking & coffee and on current smoking & total caffeine, genetic/environmental correlations __________5

Bivariate genetic models on current smoking & coffee and on current smoking & total caffeine, genetic/environmental correlations __________5

Structural equation models when analyzing caffeine as a continuous measure______________________________________________________6

*Mendelian randomization (MR) analysis*

Instrumental value of the genetic risk scores utilized in MR analyses______________________________________________________________8

MR analyses testing causal effects _________________________________________________________________________________________9

Associations between the genetic risk scores and confounding variables _________________________________________________________10

MR analyses at all time-points of ALSPAC (testing of instrumental value and causal effects) __________________________________________11

**Table S1.** Prevalences of current smoking, high coffee use and high total caffeine use as estimated in structural equation models

|  | **Male** |  |  | **Female** |  |  |  | **Male** |  |  | **Female** |  |  |
| --- | --- | --- | --- | --- | --- | --- | --- | --- | --- | --- | --- | --- | --- |
|  | **MZ** | **DZ** | **DOS** | **MZ** | **DZ** | **DOS** |  | **MZ** | **DZ** | **DOS** | **MZ** | **DZ** | **DOS** |
| *High coffee use* |  |  |  |  |  |  | *Current smoking* |  |  |  |  |  |  |
| <20 years | 2.0% | 3.2% | 2.6% | 2.4% | 2.4% | 2.6% | <20 years | 22.1% | 22.7% | 26.8% | 16.4% | 19.8% | 21.5% |
| 20-24 years | 3.4% | 5.3% | 4.4% | 4.9% | 4.9% | 5.2% | 20-24 years | 20.9% | 21.5% | 25.5% | 15.6% | 18.9% | 20.6% |
| 25-34 years | 5.6% | 8.2% | 6.9% | 9.0% | 9.0% | 9.5% | 25-34 years | 19.8% | 20.3% | 23.2% | 14.9% | 18.1% | 19.8% |
| 35-44 years | 8.7% | 12.3% | 10.6% | 15.4% | 15.4% | 16.1% | 35-44 years | 18.7% | 19.2% | 23.0% | 14.2% | 17.4% | 18.9% |
| 45-54 years | 12.9% | 17.6% | 15.4% | 24.2% | 24.2% | 25.1% | 45-54 years | 17.6% | 18.1% | 21.8% | 13.6% | 16.6% | 18.1% |
| >=55 years | 18.4% | 24.2% | 21.5% | 35.2% | 35.2% | 36.3% | >=55 years | 16.6% | 17.1% | 20.6% | 12.9% | 15.9% | 17.4% |
| *High total caffeine use* |  |  |  |  |  |  | *Current smoking* | |  |  |  |  |  |
| <20 years | 2.2% | 3.1% | 2.8% | 1.8% | 1.6% | 1.5% | <20 years | 15.4% | 16.1% | 21.5% | 12.1% | 14.9% | 16.6% |
| 20-24 years | 3.8% | 5.3% | 4.8% | 3.7% | 3.4% | 3.2% | 20-24 years | 15.2% | 15.9% | 21.2% | 11.9% | 14.7% | 16.4% |
| 25-34 years | 6.3% | 8.4% | 7.6% | 6.9% | 6.4% | 6.2% | 25-34 years | 14.9% | 15.6% | 20.9% | 11.7% | 14.5% | 16.1% |
| 35-44 years | 9.9% | 12.7% | 11.7% | 12.1% | 11.3% | 10.9% | 35-44 years | 14.7% | 15.4% | 20.6% | 11.5% | 14.2% | 15.9% |
| 45-54 years | 14.7% | 18.4% | 17.1% | 19.5% | 18.4% | 17.9% | 45-54 years | 14.5% | 15.2% | 20.3% | 11.3% | 14.0% | 15.6% |
| >=55 years | 20.9% | 25.5% | 23.9% | 29.1% | 27.8% | 27.1% | >=55 years | 14.2% | 14.9% | 20.1% | 11.1% | 13.8% | 15.4% |

MZ = monozygotic, DZ = dizygotic, DOS = dizygotic opposite sex.

**Table S2.** Twin correlations from structural equation models before constraining correlations across gender

|  | **Cross-twin within-trait** | |  |  |
| --- | --- | --- | --- | --- |
|  | **Current smoking** | **Coffee** | **Within-twin cross-trait** | **Cross-twin cross-trait** |
| MZM | 0.77 (0.65 to 0.85) | 0.50 (0.30 to 0.67) | 0.36 (0.24 to 0.48) | 0.23 (0.08 to 0.38) |
| DZM | 0.56 (0.37 to 0.72) | 0.05 (-0.28 to 0.36) | 0.34 (0.21 to 0.47) | 0.16 (-0.03 to 0.35) |
| MZF | 0.75 (0.67 to 0.81) | 0.52 (0.43 to 0.60) | 0.37 (0.30 to 0.43) | 0.27 (0.19 to 0.34) |
| DZF | 0.40 (0.25 to 0.54) | 0.28 (0.13 to 0.43) | 0.44 (0.35 to 0.52) | 0.19 (0.07 to 0.31) |
| DOS | 0.35 (0.21 to 0.48) | 0.43 (0.28 to 0.56) | 0.45 (0.38 to 0.52) | 0.25 (0.15 to 0.36) |
|  | **Cross-twin within-trait** | |  |  |
|  | **Current smoking** | **Total caffeine** | **Within-twin cross-trait** | **Cross-twin cross-trait** |
| MZM | 0.72 (0.53 to 0.85) | 0.48 (0.25 to 0.67) | 0.30 (0.15 to 0.43) | 0.20 (-0.00 to 0.38) |
| DZM | 0.55 (0.27 to 0.76) | 0.21 (-0.18 to 0.55) | 0.25 (0.07 to 0.41) | 0.20 (-0.08 to 0.44) |
| MZF | 0.73 (0.63 to 0.81) | 0.47 (0.36 to 0.58) | 0.20 (0.11 to 0.29) | 0.17 (0.07 to 0.28) |
| DZF | 0.46 (0.26 to 0.63) | 0.21 (-0.02 to 0.42) | 0.41 (0.29 to 0.51) | 0.18 (-0.00 to 0.34) |
| DOS | 0.27 (0.06 to 0.46) | 0.42 (0.20 to 0.61) | 0.26 (0.16 to 0.36) | 0.20 (0.03 to 0.36) |

MZM = monozygotic male twin pairs, DZM = dizygotic male twin pairs, MZF = monozygotic female twin pairs, DZF = dizygotic female twin pairs,

DOS = dizygotic opposite sex twin pairs.

**
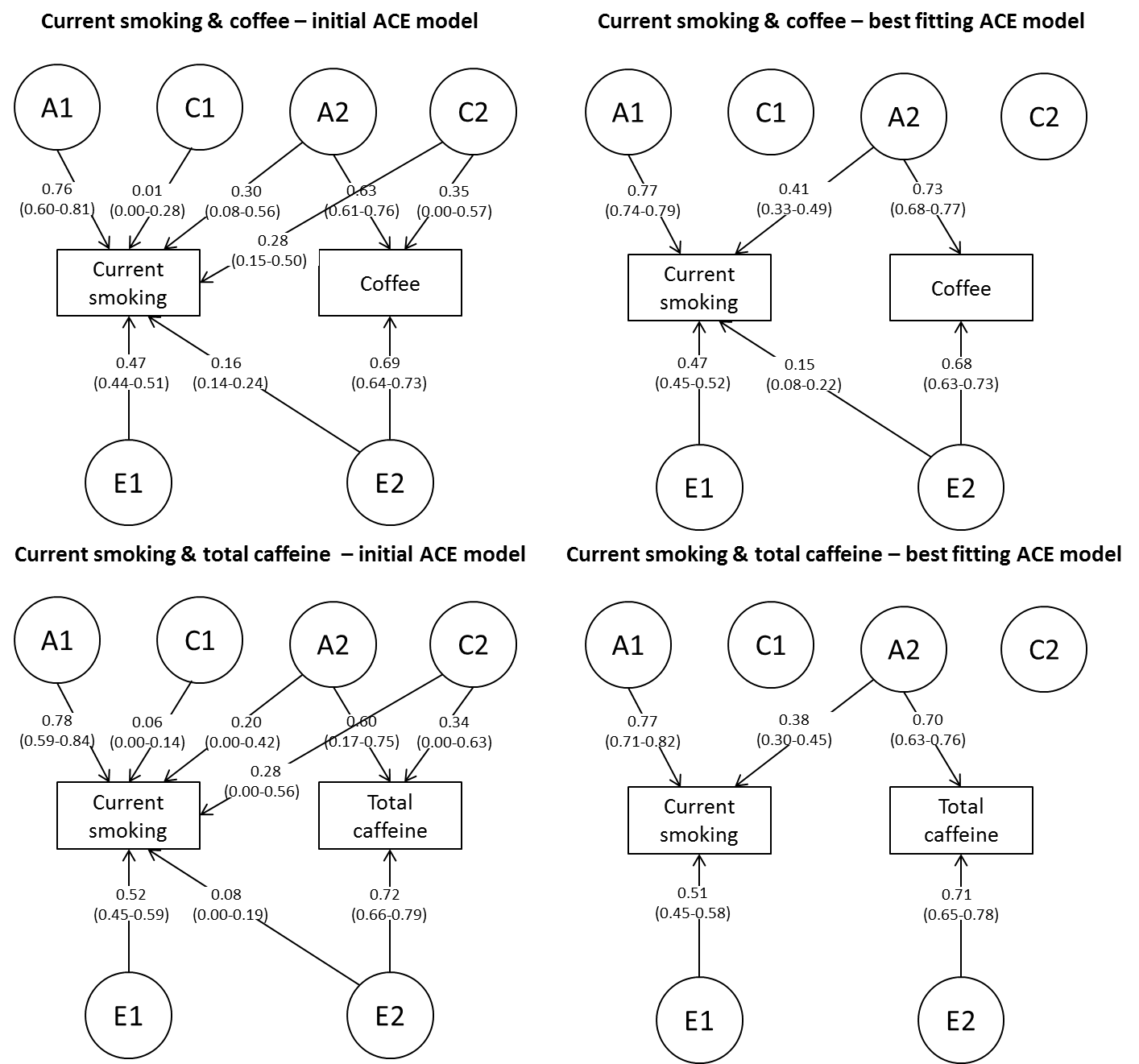

Figure S1.** Path estimates for bivariate genetic models on current smoking & coffee and on current smoking & total caffeine. A = additive genetic factors, C = common genetic factors, E = unique environmental factors. Both the initial models and the best-fitting models are shown.

**
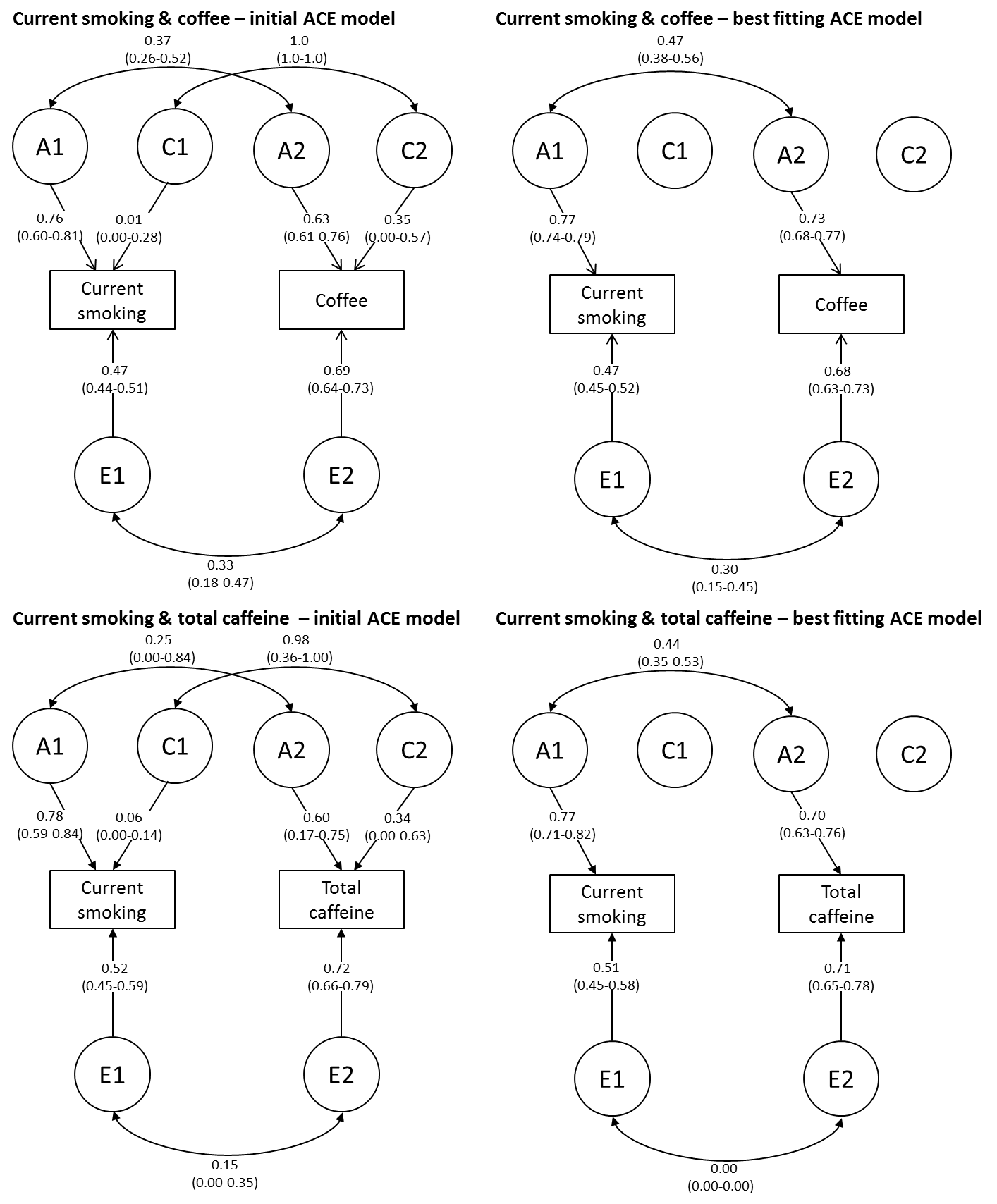
**

**Figure S2.** Path estimates for bivariate genetic models on current smoking & coffee and on current smoking & total caffeine. A = additive genetic factors, C = common genetic factors, E = unique environmental factors. Here, genetic (between A1 and A2), common environmental (between C1 and C2) and unique environmental (E1 and E2) correlations are shown. Calculation of the genetic correlation was based on the following formula: a_CurrentSmoking_ * a_CurrentSmoking-Coffee_ / √(a^2^_CurrentSmoking_) * √( a^2^_CurrentSmoking-Coffee_ + a^2^_Coffee_), where a_CurrentSmoking_ and a_Coffee_ represent the path loadings going from A1 to ‘Current smoking’ and from A2 to ‘Coffee’, respectively and a_CurrentSmoking-Coffee_ represents the path loading going from A2 to ‘Current smoking’ in Figure S1. Environmental correlations were calculated in the same way. Both the initial models and the best-fitting models are shown.

**Table S2.** Structural equation models to explore additive genetic (A), common environmental (C) and unique environmental (E) influences on current smoking and caffeine use in mg per day, and on their overlap

| **A: Current smoking and coffee use (N=10,368)** | **Estimated parameters** | **-2LL** | **df** | **Compared with** | **X²** | **P-value** |
| --- | --- | --- | --- | --- | --- | --- |
| 1. Saturated five-group model | 48 | 142675.11 | 20688 | - | - | - |
| 2. β’s covariate dropped | 44 | 144264.91 | 20692 | 1 | 1589.8 | <0.001 |
| 3. Thresholds/β’s constrained across sex for smoking | 43 | 142695.31 | 20693 | 1 | 20.19 | <0.001 |
| 4. Means/β’s constrained across sex for coffee | 42 | 143896.88 | 20694 | 1 | 1221.77 | <0.001 |
| 5. Cross-trait-within twin correlation constrained across twin birth order | 43 | 142679.51 | 20693 | 1 | 4.39 | 0.49 |
| 6. Cross-trait-cross twin correlation constrained across twin birth order | 38 | 142688.03 | 20698 | 5 | 8.52 | 0.13 |
| **7. Correlations MZM=MZF + correlations DZM=DZF=DOS** | **26** | **142700.75** | **20710** | **6** | **12.72** | **0.39** |
| 8. ACE model | 25 | 143238.14 | 20716 | 1 | 563.03 | <0.001 |
| 9. C for coffee dropped | 24 | 143238.18 | 20717 | 8 | 0.04 | 0.84 |
| 10. C for smoking dropped | 23 | 143238.18 | 20718 | 9 | 0 | 1.0 |
| **11. C for overlap dropped** | **22** | **143238.51** | **20719** | **10** | **0.33** | **0.57** |
| 12. A for coffee dropped | 21 | 143830.97 | 20720 | 11 | 592.46 | <0.001 |
| 13. A for smoking dropped | 21 | 143500.17 | 20720 | 11 | 261.66 | <0.001 |
| 14. A for overlap dropped | 21 | 143399.58 | 20720 | 11 | 161.07 | <0.001 |
| 15. E for overlap dropped | 21 | 143257.08 | 20720 | 11 | 18.57 | <0.001 |
| **B: Current smoking and total caffeine use (N=8,060)** | **Estimated parameters** | **-2LL** | **df** | **Compared with** | **X²** | **P-value** |
| 1. Saturated five-group model | 48 | 110141.85 | 16072 | - | - | - |
| 2. β’s covariate dropped | 44 | 111708.68 | 16076 | 1 | 1566.83 | <0.001 |
| 3. Thresholds/β’s constrained across sex for smoking | 43 | 110157.3 | 16077 | 1 | 15.45 | 0.01 |
| 4. Means/β’s constrained across sex for total caffeine | 42 | 110675.94 | 16078 | 1 | 534.09 | <0.001 |
| 5. Cross-trait-within twin correlation constrained across twin birth order | 43 | 110144.57 | 16077 | 1 | 2.72 | 0.74 |
| 6. Cross-trait-cross twin correlation constrained across twin birth order | 38 | 110155.32 | 16082 | 5 | 10.75 | 0.06 |
| **7. Correlations MZM=MZF + correlations DZM=DZF=DOS** | **26** | **110166.48** | **16094** | **6** | **11.16** | **0.52** |
| 8. ACE model | 25 | 110391.34 | 16100 | 1 | 249.49 | <0.001 |
| 9. C for total caffeine dropped | 24 | 110391.34 | 16101 | 8 | 0 | 1.0 |
| 10. C for smoking dropped | 23 | 110391.34 | 16102 | 9 | 0 | 1.0 |
| **11. C for overlap dropped** | **22** | **110391.8** | **16103** | **10** | **0.46** | **0.5** |
| 12. A for total caffeine dropped | 21 | 110751.44 | 16104 | 11 | 359.64 | <0.001 |
| 13. A for smoking dropped | 21 | 110541.96 | 16104 | 11 | 150.16 | <0.001 |
| 14. A for overlap dropped | 21 | 110456.08 | 16104 | 11 | 64.28 | <0.001 |
| 15. E for overlap dropped | 21 | 110400.39 | 16104 | 11 | 8.59 | <0.001 |

MZM = monozygotic male twin pairs, MZF = monozygotic female twin pairs, DZM = dizygotic male twin pairs, DZF = dizygotic female twin pairs, DOS = dizygotic opposite sex twin pairs. A threshold represents the prevalence of smoking. β = effect of age on the prevalence (threshold) of smoking or mean coffee/caffeine use. The best fitting models are depicted in bold.

|  | **Cross-twin within-trait** | |  |  | **Cross-twin within-trait** | |  |  |
| --- | --- | --- | --- | --- | --- | --- | --- | --- |
|  | **Current smoking** | **Coffee** | **Within-twin cross-trait** | **Cross-twin cross-trait** | **Current smoking** | **Total caffeine** | **Within-twin cross-trait** | **Cross-twin cross-trait** |
| MZ | 0.75 (0.69 to 0.80) | 0.46 (0.43 to 0.49) | 0.29 (0.25 to 0.33) | 0.23 (0.19 to 0.28) | 0.72 (0.64 to 0.79) | 0.43 (0.39 to 0.47) | 0.21 (0.17 to 0.26) | 0.17 (0.12 to 0.23) |
| DZ | 0.41 (0.32 to 0.49) | 0.22 (0.17 to 0.27) | 0.35 (0.32 to 0.38) | 0.14 (0.09 to 0.19) | 0.39 (0.26 to 0.51) | 0.21 (0.14 to 0.27) | 0.31 (0.27 to 0.35) | 0.09 (0.02 to 0.16) |

**Table S3.** Twin correlations for current smoking and coffee use in mg per day (N=10,368) and for current smoking and total caffeine use in mg per day (N=8,060) from the best-fitting saturated models

MZ = monozygotic, DZ = dizygotic. Cross-twin within trait = correlation between smoking twin 1 and smoking twin 2 or coffee/caffeine twin 1 and coffee/caffeine twin 2. Within-twin cross-trait = correlation between smoking and coffee/caffeine in one twin. Cross-twin cross-trait = correlation between smoking twin 1 and coffee/caffeine twin 2.

**Table S4.** Estimates of additive genetic (A), common environmental (C) and unique environmental (E) influences on current smoking and coffee use in mg per day (N=10,368) and on current smoking and total caffeine use in mg per day (N=8,060), from the full and the best-fitting bivariate genetic models

|  | **Current smoking** | **Coffee** | **Phenotypic overlap** | **Current smoking** | **Total caffeine** | **Phenotypic overlap** |
| --- | --- | --- | --- | --- | --- | --- |
| *Full model* |  |  |  |  |  |  |
| A | 0.69 (0.49 to 0.80) | 0.47 (0.42 to 0.50) | 0.78 (0.54 to 0.89) | 0.63 (0.35 to 0.79) | 0.45 (0.35 to 0.48) | 0.77 (0.61 to 0.92) |
| C | 0.06 (0.00 to 0.23) | 0.00 (0.00 to 0.04) | 0.02 (0.00 to 0.22) | 0.09 (0.00 to 0.33) | 0.00 (0.00 to 0.08) | 0.00 (0.00 to 0.27) |
| E | 0.25 (0.20 to 0.31) | 0.53 (0.49 to 0.55) | 0.20 (0.11 to 0.30) | 0.28 (0.21 to 0.36) | 0.55 (0.52 to 0.59) | 0.23 (0.08 to 0.39) |
| *Best-fitting model* |  |  |  |  |  |  |
| A | 0.76 (0.70 to 0.81) | 0.48 (0.44 to 0.50) | 0.80 (0.72 to 0.87) | 0.73 (0.65 to 0.80) | 0.45 (0.41 to 0.48) | 0.77 (0.62 to 0.92) |
| C | - | - | - | - | - | - |
| E | 0.24 (0.20 to 0.30) | 0.52 (0.50 to 0.55) | 0.20 (0.11 to 0.30) | 0.27 (0.21 to 0.35) | 0.55 (0.48 to 0.59) | 0.23 (0.08 to 0.39) |
| Genetic correlation | 0.42 (0.37 to 0.48) | |  | 0.36 (0.28 to 0.44) | |  |
| Unique environmental correlation | 0.18 (0.10 to 0.26) | |  | 0.15 (0.05 to 0.26) | |  |

Phenotypic overlap reflects how much of the observational association between current smoking and coffee/total caffeine is due to additive genetic (A), common environmental (C) and unique environmental (E) influences, summing up to 1.

**
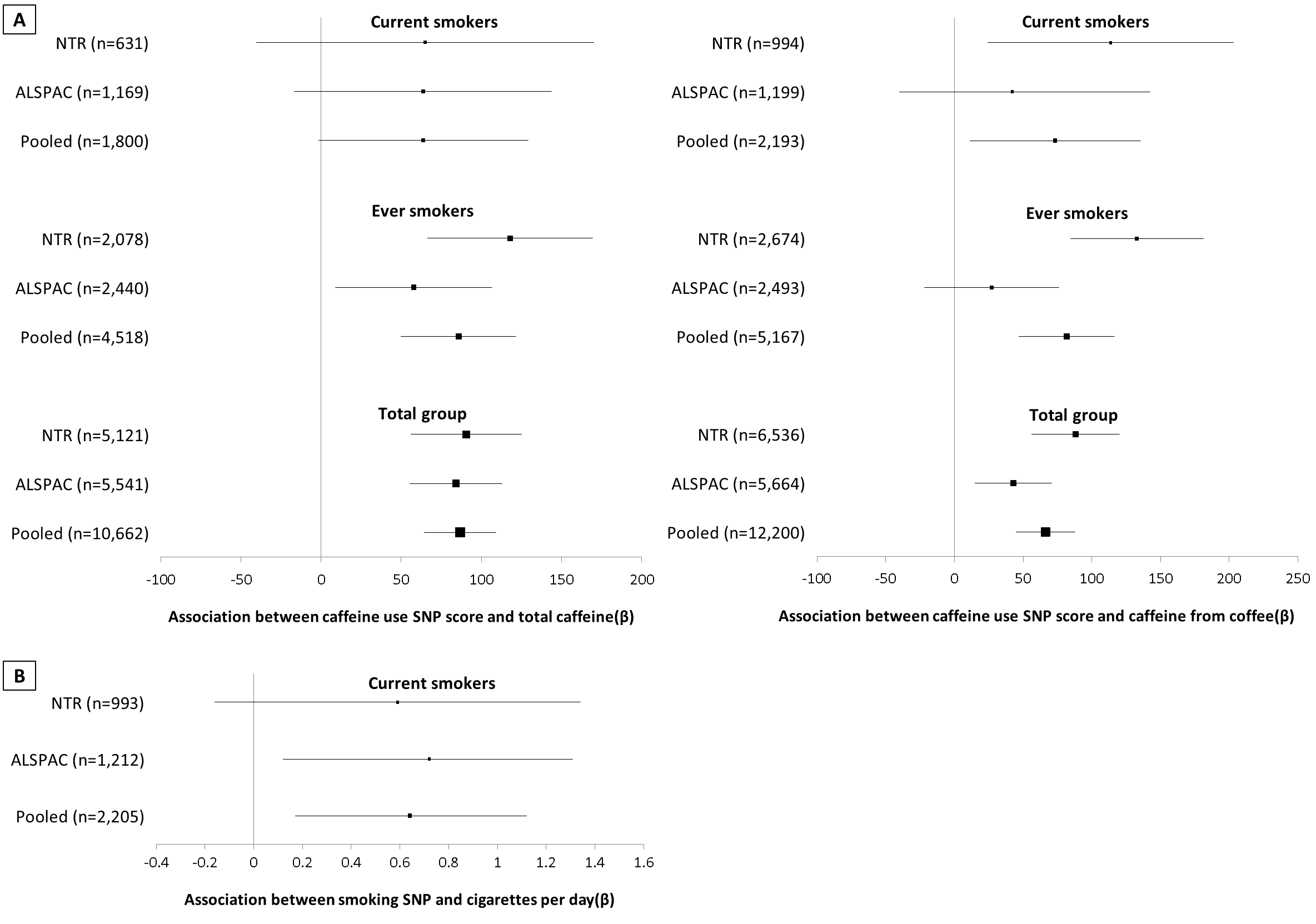
**

**Figure S3.** Instrumental value of the genetic risk scores. The forest plots show associations between the caffeine use SNP score and total caffeine use and caffeine from coffee in mg per day (A) and between the smoking SNP and cigarettes smoked per day (B). NTR = Netherlands Twin Register; ALSPAC = Avon Longitudinal Study of Parents and Children.

**
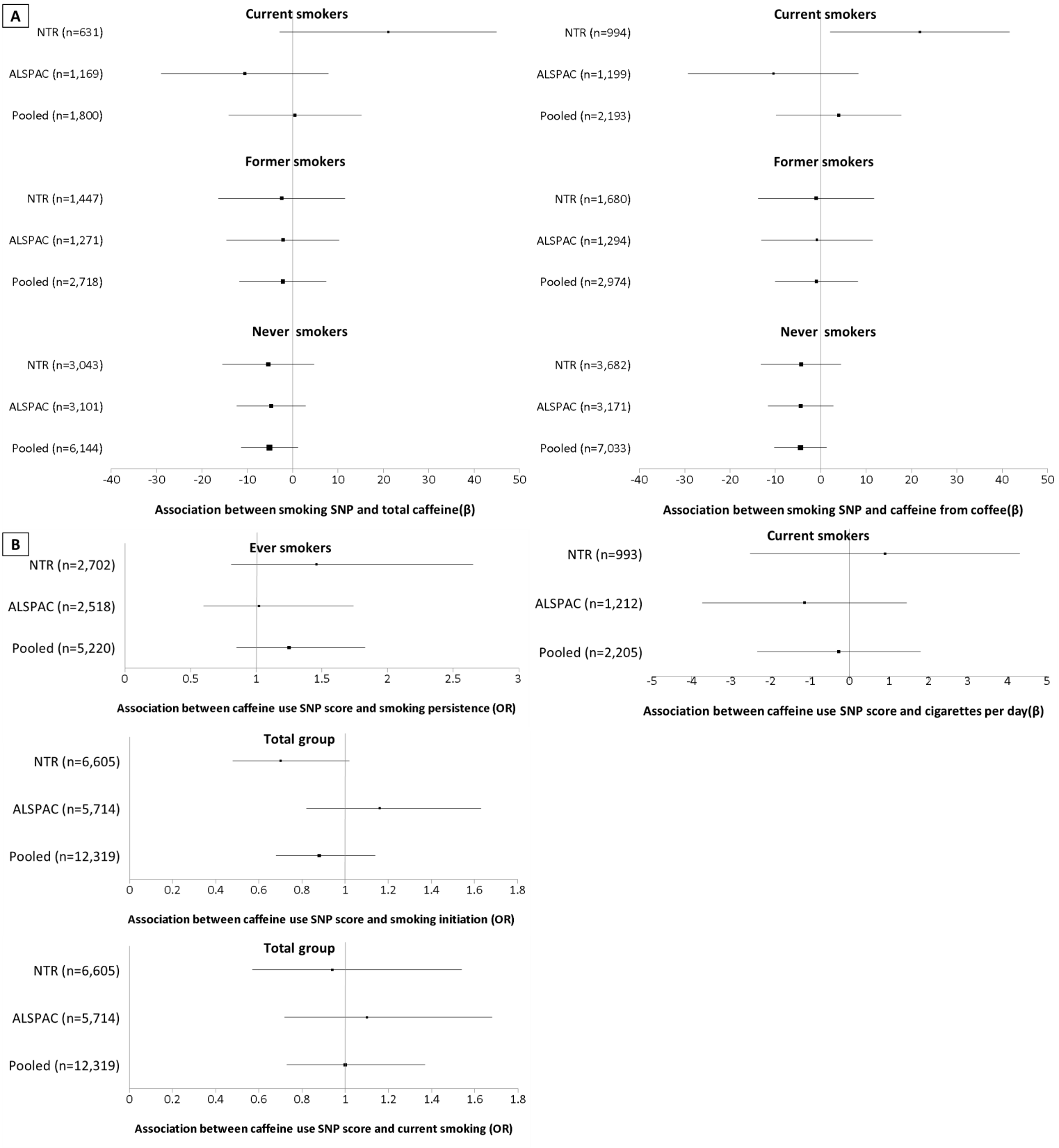
**

**Figure S4.** MR analyses testing causal effects. The forest plots show associations between the smoking SNP and total caffeine use and caffeine from coffee in mg per day (A) and between the caffeine use SNP score and smoking behaviour (cigarettes smoked per day, smoking persistence, smoking initiation and current smoking) (B). NTR = Netherlands Twin Register; ALSPAC = Avon Longitudinal Study of Parents and Children.

**Table S5.** Associations between the caffeine use SNP score and the smoking SNP and confounding variables in *the Netherlands Twin Register (NTR)* and the *Avon Longitudinal Study of Parents and Children (ALSPAC)*

|  |  | **Education**  **– OR (95% CI)** | **Social class**  **– OR (95% CI)** |
| --- | --- | --- | --- |
| *NTR* | *N* | *5,043* |  |
|  | Caffeine use SNP score | 0.99 (0.66 to 1.50) | - |
|  | Smoking SNP | 0.95 (0.86 to 1.04) | - |
| *ALSPAC* | *N* | *5,565* | *4,814* |
|  | Caffeine use SNP score | 1.01 (0.71 to 1.42) | 0.88 (0.60 to 1.27) |
|  | Smoking SNP | 0.94 (0.87 to 1.02) | 0.92 (0.84 to 1.00) |

Logistic regression analyses were performed with education (0 = low 1 = high^[[1]](#footnote-1)^) or social class (only in ALSPAC: 0 = low, 1 = high^[[2]](#footnote-2)^), as the dependent variable and the caffeine use SNP score or the smoking SNP as the independent variable. For the NTR, analyses were corrected for family clustering by utilizing the robust cluster option in STATA.

**Table S6**. Mendelian Randomization analyses between the caffeine use SNP score and daily caffeine consumption (in mg) and smoking behaviour in the *Avon Longitudinal Study of Parents and Children (ALSPAC)*

|  |  | **Total Caffeine**  **in current smokers**  **– β (95% CI)** | **Caffeine coffee**  **in current smokers – β (95% CI)** | **Cigarettes per day**  **in current smokers – β (95% CI)** |  |  | |
| --- | --- | --- | --- | --- | --- | --- | --- |
|  | *N* | *1,283* | *1,296* | *1,284* |  |  |  |
| *18w gestation* | Caffeine use SNP score | 45.83 (-23.80 to 115.46) | 21.94 (-42.84 to 86.72) | 0.50 (-2.06 to 3.05) |  |  |  |
|  | *N* | *1,150* | *1,213* | *1,253* |  |  |  |
| *32w gestation* | Caffeine use SNP score | 98.61 (26.03 to 171.19) | 42.46 (-22.84 to 107.75) | 0.83 (-1.63 to 3.29) |  |  |  |
|  | *N* | *977* | *1,036* | *1,415* |  |  |  |
| *2 months* | Caffeine use SNP score | 100.21 (13.21 to 187.21) | 87.31 (0.83 to 173.80) | -0.39 (-2.89 to 2.11) |  |  |  |
|  | *N* | *1,169* | *1,199* | *1,212* |  |  |  |
| *47 months* | Caffeine use SNP score | 63.63 (-16.60 to 143.86) | 42.15 (-39.99 to 124.29) | -1.14 (-3.73 to 1.45) |  |  |  |
|  | *N* | *948* | *978* | *996* |  |  |  |
| *85 months* | Caffeine use SNP score | -28.52 (-120.78 to 63.74) | -31.41 (-131.04 to 68.23) | -3.12 (-6.27 to 0.02) |  |  |  |
|  | *N* | *788* | *822* | *832* |  |  |  |
| *97 months* | Caffeine use SNP score | 47.41 (-56.29 to 151.10) | 18.74 (-90.47 to 127.96) | 0.55 (-3.03 to 4.12) |  |  |  |
|  | *N* | *449* | *550* | *716* |  |  |  |
| *145 months* | Caffeine use SNP score | -18.20 (-133.61 to 97.22) | -9.33 (-121.16 to 102.49) | 0.73 (-3.20 to 4.65) |  |  |  |
|  |  | **Total Caffeine**  **in ever smokers**  **– β (95% CI)** | **Caffeine coffee**  **in ever smokers – β (95% CI)** |  |  |  |  |
|  | *N* | *3,555* | *3,587* |  |  |  |  |
| *18w gestation* | Caffeine use SNP score | 60.31 (22.74 to 97.88) | 43.36 (9.96 to 76.75) |  |  |  |  |
|  | *N* | *3,002* | *3,124* |  |  |  |  |
| *32w gestation* | Caffeine use SNP score | 73.62 (33.50 to 113.74) | 30.10 (-4.30 to 64.50) |  |  |  |  |
|  | *N* | *2,181* | *2,293* |  |  |  |  |
| *2 months* | Caffeine use SNP score | 71.81 (18.16 to 125.45) | 59.07 (8.42 to 109.73) |  |  |  |  |
|  | *N* | *2,440* | *2,493* |  |  |  |  |
| *47 months* | Caffeine use SNP score | 57.80 (8.89 to 106.72) | 27.02 (-21.85 to 75.89) |  |  |  |  |
|  | *N* | *2,072* | *2,116* |  |  |  |  |
| *85 months* | Caffeine use SNP score | 37.53 (-17.47 to 92.52) | 4.71 (-51.87 to 61.29) |  |  |  |  |
|  | *N* | *2,035* | *2,103* |  |  |  |  |
| *97 months* | Caffeine use SNP score | 70.77 (16.58 to 124.96) | 44.67 (-10.95 to 100.28) |  |  |  |  |
|  | *N* | *1,188* | *1,351* |  |  |  |  |
| *145 months* | Caffeine use SNP score | 17.96 (-46.40 to 82.32) | -2.81 (-63.14 to 57.51) |  |  |  |  |
|  |  | **Total Caffeine**  **in total group**  **– β (95% CI)** | **Caffeine coffee**  **in total group – β (95% CI)** | **Smoking initiation in total group**  **– OR (95% CI)** | **Smoking persistence in total group**  **– OR (95% CI)** | **Current smoking in total group – OR (95% CI)** |  |
|  | *N* | *7,300* | *7,345* | *7,387* | *3,610* | *7,387* |  |
| *18w gestation* | Caffeine use SNP score | 53.07 (29.81 to 76.34) | 28.77 (8.65 to 48.89) | 1.11 (0.82 to 1.50) | 1.28 (0.81 to 2.01) | 1.26 (0.84 to 1.88) |  |
|  | *N* | *6,233* | *6,474* | *6,615* | *3,206* | *6,615* |  |
| *32w gestation* | Caffeine use SNP score | 68.94 (44.69 to 93.19) | 31.20 (10.81 to 51.58) | 1.15 (0.84 to 1.59) | 1.38 (0.85 to 2.23) | 1.34 (0.89 to 2.02) |  |
|  | *N* | *4,861* | *5,084* | *6,876* | *3,076* | *6,876* |  |
| *2 months* | Caffeine use SNP score | 83.75 (51.82 to 115.68) | 56.01 (26.96 to 85.05) | 1.26 (0.92 to 1.73) | 1.55 (0.95 to 2.51) | 1.54 (1.04 to 2.28) |  |
|  | *N* | *5,541* | *5,664* | *5,714* | *2,518* | *5,714* |  |
| *47 months* | Caffeine use SNP score | 84.22 (55.49 to 112.94) | 42.94 (15.05 to 70.82) | 1.16 (0.82 to 1.63) | 1.02 (0.60 to 1.74) | 1.10 (0.72 to 1.68) |  |
|  | *N* | *4,959* | *5,066* | *5,134* | *2,147* | *5,134* |  |
| *85 months* | Caffeine use SNP score | 71.23 (40.51 to 101.96) | 26.73 (-3.95 to 57.40) | 1.30 (0.90 to 1.88) | 0.94 (0.52 to 1.68) | 1.13 (0.71 to 1.80) |  |
|  | *N* | *4,622* | *4,776* | *4,848* | *2,133* | *4,848* |  |
| *97 months* | Caffeine use SNP score | 84.43 (52.40 to 116.47) | 42.73 (11.20 to 74.25) | 1.36 (0.93 to 1.97) | 1.17 (0.64 to 2.13) | 1.33 (0.80 to 2.19) |  |
|  | *N* | *2,872* | *3,235* | *4,375* | *1,761* | *4,375* |  |
| *145 months* | Caffeine use SNP score | 60.06 (23.57 to 96.56) | 25.27 (-8.12 to 58.66) | 1.29 (0.86 to 1.92) | 1.89 (0.98 to 3.66) | 1.91 (1.11 to 3.27) |  |

Linear/logistic regression analyses were performed with total caffeine use, caffeine use through coffee only, cigarettes per day, smoking initiation (0 = never smoking, 1 = ever smoking) or smoking cessation (0 = former smoking, 1 = current smoking) as the dependent variable and the caffeine use SNP score as the independent variable. All analyses were adjusted for age (continuous).

**Table S7**. Mendelian Randomization analyses between the smoking SNP and smoking behaviour and daily caffeine consumption (in mg) in the *Avon Longitudinal Study of Parents and Children (ALSPAC)*

|  |  | **Current smokers** | | | **Former smokers** | | **Never smokers** | |
| --- | --- | --- | --- | --- | --- | --- | --- | --- |
|  |  | **Cigarettes per day**  **– β (95% CI)** | **Total caffeine**  **– β (95% CI)** | **Caffeine coffee**  **– β (95% CI)** | **Total caffeine**  **– β (95% CI)** | **Caffeine coffee**  **– β (95% CI)** | **Total caffeine**  **– β (95% CI)** | **Caffeine coffee**  **– β (95% CI)** |
|  | *N* | *1,284* | *1,283* | *1,296* | *2,272* | *2,291* | *3,745* | *3,758* |
| *18w gestation* | Smoking SNP | 0.23 (-0.34 to 0.81) | 9.43 (-6.32 to 25.18) | 4.35 (-10.25 to 18.94) | 2.18 (-7.47 to 11.82) | 4.01 (-4.39 to 12.40) | 0.92 (-5.00 to 6.84) | -1.25 (-6.25 to 3.74) |
|  | *N* | *1,253* | *1,150* | *1,213* | *1,852* | *1,911* | *3,231* | *3,350* |
| *32w gestation* | Smoking SNP | 0.25 (-0.30 to 0.81) | -6.41 (-22.56 to 9.74) | -8.18 (-22.78 to 6.42) | 2.89 (-6.75 to 12.52) | 4.32 (-3.80 to 12.44) | -1.76 (-7.89 to 4.37) | -3.33 (-8.45 to 1.78) |
|  | *N* | *1,415* | *977* | *1,036* | *1,204* | *1,257* | *2,680* | *2,791* |
| *2 months* | Smoking SNP | 0.79 (0.23 to 1.36) | -2.61 (-22.02 to 16.80) | -0.80 (-20.13 to 18.53) | 12.84 (-1.79 to 27.46) | 12.55 (-0.29 to 25.39) | -6.65 (-15.14 to 1.85) | -7.07 (-14.43 to 0.30) |
|  | *N* | *1,212* | *1,169* | *1,199* | *1,271* | *1,294* | *3,101* | *3,171* |
| *47 months* | Smoking SNP | 0.72 (0.12 to 1.31) | -10.51 (-28.97 to 7.94) | -10.47 (-29.26 to 8.33) | -2.12 (-14.53 to 10.30) | -0.84 (-13.09 to 11.42) | -4.74 (-12.34 to 2.87) | -4.42 (-11.60 to 2.75) |
|  | *N* | *996* | *948* | *978* | *1,124* | *1,138* | *2,887* | *2,950* |
| *85 months* | Smoking SNP | 0.99 (0.30 to 1.67) | 23.52 (3.31 to 43.73) | 12.29 (-9.52 to 34.10) | -9.30 (-23.30 to 4.69) | -7.81 (-21.31 to 5.69) | -3.97 (-11.73 to 3.79) | -7.42 (-14.95 to 0.11) |
|  | *N* | *832* | *788* | *822* | *1,247* | *1,281* | *2,587* | *2,673* |
| *97 months* | Smoking SNP | 1.26 (0.47 to 2.04) | 1.50 (-21.22 to 24.23) | -2.41 (-26.35 to 21.54) | -3.11 (-16.39 to 10.16) | -3.58 (-16.87 to 9.71) | 1.49 (-7.15 to 10.12) | 1.48 (-6.64 to 9.60) |
|  | *N* | *716* | *449* | *550* | *739* | *801* | *1,684* | *1,884* |
| *145 months* | Smoking SNP | 1.39 (0.51 to 2.28) | 16.16 (-11.14 to 43.46) | -8.80 (-34.42 to 16.81) | 8.21 (-8.34 to 24.75) | 4.09 (-10.62 to 18.79) | 2.54 (-7.33 to 12.40) | -3.01 (-11.59 to 5.58) |

Linear regression analyses were performed with cigarettes per day, total caffeine use or caffeine use through coffee only as the dependent variable and the smoking SNP as the independent variable, stratified on smoking status. All analyses were adjusted for age (continuous).

1. For NTR low = primary school only, lower vocational schooling, lower secondary schooling, intermediate vocational schooling or intermediate/higher secondary schooling and high = higher vocational schooling or university; for ALSPAC low = Secondary Education (CSE), vocational or O level and high = A level or Degree. [↑](#footnote-ref-1)
2. Low = class II (non-manual), class III (manual), class IV or class V and high = class I or class II. [↑](#footnote-ref-2)
